# Stage-Wise Learning of Reaching Using Little Prior Knowledge

**DOI:** 10.3389/frobt.2018.00110

**Published:** 2018-10-01

**Authors:** François de La Bourdonnaye, Céline Teulière, Jochen Triesch, Thierry Chateau

**Affiliations:** ^1^CNRS, SIGMA Clermont, Institut Pascal, Université Clermont Auvergne, Clermont-Ferrand, France; ^2^Frankfurt Institute for Advanced Studies, Frankfurt am Main, Germany

**Keywords:** deep reinforcement learning, weakly-supervised, stage-wise learning, manipulation robotics, hierarchical learning

## Abstract

In some manipulation robotics environments, because of the difficulty of precisely modeling dynamics and computing features which describe well the variety of scene appearances, hand-programming a robot behavior is often intractable. Deep reinforcement learning methods partially alleviate this problem in that they can dispense with hand-crafted features for the state representation and do not need pre-computed dynamics. However, they often use prior information in the task definition in the form of shaping rewards which guide the robot toward goal state areas but require engineering or human supervision and can lead to sub-optimal behavior. In this work we consider a complex robot reaching task with a large range of initial object positions and initial arm positions and propose a new learning approach with minimal supervision. Inspired by developmental robotics, our method consists of a weakly-supervised stage-wise procedure of three tasks. First, the robot learns to fixate the object with a 2-camera system. Second, it learns hand-eye coordination by learning to fixate its end-effector. Third, using the knowledge acquired in the previous steps, it learns to reach the object at different positions and from a large set of initial robot joint angles. Experiments in a simulated environment show that our stage-wise framework yields similar reaching performances, compared with a supervised setting without using kinematic models, hand-crafted features, calibration parameters or supervised visual modules.

## 1. Introduction

In manipulation robotics, various tasks cannot be programmed by hand because dynamics is hard to compute or/and hand-crafted features do not describe well enough the variety of scene appearances. Deep reinforcement learning tackles both of these issues in that features are automatically computed by optimization and dynamics is not required (Levine et al., 2016; Gu et al., 2017; Riedmiller et al., 2018). In manipulation robotics, the success of a task is often defined by a sparse reward (i.e., a positive signal is given to the robot only if the full task is successfully completed) in a high-dimensional state space, which makes learning slow since in some high-dimensional robotics tasks, it is very unlikely to get a first success when the initial states are far from the targeted ones. Although the use of several agents in parallel has shown good performances with a sparse only reward (Levine et al., 2017), it requires expensive resources and materials as well as a simplified action space which are not always possible to get. Provided an expert knowledge is available, learning by demonstration (Kober and Peters, 2009; Nair et al., 2017; Sermanet et al., 2017) can also be used to guide the robot to sparse-reward areas. But it requires prior knowledge on the optimal/sub-optimal behavior for a specific task.

An alternative solution consists of using shaping rewards. They allow to guide the exploration of the agent toward goal state areas, i.e., the probability of receiving sparse rewards is increased. Using shaping rewards leads to two main issues. First, they can lead to sub-optimal policies (Popov et al., 2017) by biasing the exploration process, e.g., the solution specified by the reward function may not be optimal. Second, they generally require tedious engineering work or other forms of supervision. For instance, for manipulation tasks such as block stacking, reaching, door pushing or pulling (Deisenroth et al., 2011; Chebotar et al., 2017; Ghadirzadeh et al., 2017; Gu et al., 2017; Tsurumine et al., 2017), an informative reward is computed based on a distance measure between a current and a target pose. However, this requires to know robot kinematics and target position (through supervised visual tracking or measure). In a similar way, in Levine et al. (2015, 2016) (for tasks such as placing wooden rings or screwing bottle caps onto bottles), informative shaping rewards have been computed using a distance measure between current end-effector or manipulated object positions and their corresponding target positions. However, they require knowledge of kinematics or non-trivial visual modules. For similar tasks, a more sophisticated set-up has been proposed in Finn et al. (2016): the shaping reward is based on the distance between current visual features and target features, both of them being computed by an autoencoder. This requires to place the robot at the target position and extract target visual features each time the target location changes.

Another category of solutions to make learning with sparse-only rewards tractable consists in decomposing the whole problem into simpler sub-problems. For instance, assuming one goal state is known, a mechanism of learning from easy missions (Asada et al., 1996) can be used to learn very precise robotic manipulation tasks such as inserting and turning a key in a lock or assembling a gear onto an axle (Florensa et al., 2017). This method consists in starting learning the task from initial states close to the goal state and as far as learning improves, states are initialized further and further. Nevertheless, this method assumes the knowledge of a goal state and up to our knowledge, has not been proven efficient yet for a multi target position setting. Furthermore, hierarchical reinforcement learning can be used to decompose complex tasks such as block stacking into simpler sub-tasks. For instance, (Gudimella et al., 2017) quickly learns a block stacking task using Concept Network Reinforcement Learning (CNRL), a hierarchical framework which decomposes the problem into sub-problems like reaching the working area, grasping, reaching the second working area, and stacking. However, these sub-tasks use shaping rewards requiring kinematics and target pose knowledge. Besides, a similar task is learned using another hierarchical reinforcement learning framework called Scheduled Auxiliary Control (SAC-X) (Riedmiller et al., 2018). This uses auxiliary rewards (sparse for most of them) encouraging the robot to discover sub-goals such as making objects closer, making an object higher or lower than the other one, maximizing or minimizing the sum of finger tactile sensors. One key aspect of this architecture is that learning to achieve the sub-goals does not bias the learned policy and is only used to explore more the environment. However, some of these auxiliary rewards still require object tracking in the images and are not necessarily adaptable to any object.

In this paper, we consider the task of touching an object with the end-effector palm and we propose to learn it by decomposing the whole problem into simpler sub-problems and by using minimal prior knowledge. In other terms, our approach does not use kinematic models, hand-crafted features, calibration parameters and supervised visual modules. The task more precisely consists of reaching an object put on a table with the end-effector palm at several object positions and from several initial arm positions. This task can be considered and used as a pre-grasping task because target arm joint angles for our task are very close to target arm joint angles for grasping. The difficulty of our task relies on the fact that the arm has to reach from a large set of initial conditions (different object positions and initial arm positions, see Figure 5) so that it frequently has to substantially modify its orientation to reach the target with the palm. In this paper, we extend our prior work de La Bourdonnaye et al. (2018) to the more complex setting of multiple object positions. Besides, we conduct additional experiments to study of the influence of different reward terms. In this work, we have taken inspiration from the human development (Fischer, 1980; Carey et al., 1997) and developmental robotics (Hoffmann et al., 2005). To grasp an object, humans usually fixate it first, and then grasp it. This assertion does not mean that the only way to localize an object is to bring it in the fovea. Indeed, expert jugglers use information in the periphery of vision to detect juggling balls (Huys and Beek, 2002) and a monkey study reported that 81% of the neurons of the parietal reach region encode location in eye-centered coordinates (Batista et al., 1999). However, it can be a sufficient tool if these neurons are deficient (in case of widespread cortical atrophy Carey et al., 1997) and has the advantage of being compact. The rationale of our method is that an informative shaping reward for the object touching task can be constructed from the knowledge of simpler anterior tasks learned with minimal supervision. More precisely, the robot first learns to fixate objects (de La Bourdonnaye et al., 2017) and its own end-effector using a single deep reinforcement learning framework with little prior knowledge in the goal specification. Based on these two skills, an informative shaping reward is built, efficiently guiding the robot toward goal state areas. Our experiments show that learning this task with our weakly-supervised stage-wise framework yields same reaching performances as with a supervised reward, while learning with a sparse reward is slow. Our contribution is the design of our weakly-supervised framework which is efficient to learn to reach objects at several object positions and from several initial arm positions in a single shot.

The remainder is organized as follows. Section 2 presents basics about deep reinforcement learning, our stage-wise framework for reaching learning and the experimental protocol designed to validate our framework. Section 3 describes the results obtained and section 4 discusses the work from a broader perspective.

## 2. Methods and materials

This section presents the methods and the materials used in our experiments.

### 2.1. Background

Our work uses deep reinforcement learning. This section provides basics of reinforcement learning and the algorithm used to learn the different stages.

#### 2.1.1. Reinforcement learning

Reinforcement learning (RL) is a class of algorithms used to solve sequential decision making problems through learning. It is distinguishable from the dynamic programming category in that it does not require prior knowledge about dynamics and the reward signal. Most RL algorithms are based on Markov decision processes < *S, A, R, T* > where S is the set of states, A the set of actions, T the transition model (*T* : *S* × *A* → *S*) and R the reward function (*R* : *S* × *A* → ℝ).

The source of learning comes from interaction between the agent and the environment and is composed of tuples < ***s***,***a***, *r*,***s***^**′**^> called transitions. ***s*** represents a state value and ***a*** the action performed at state ***s***. After the execution of the action ***a***, the agent receives a reward *r* and reaches a new state ***s***^**′**^.

The goal of an RL agent is to adapt its behavior to maximize a criterion linked to the future rewards. In the paper, we consider the sum of discounted future rewards as a learning goal: $J=\overset{\infty }{\underset{k=0}{\sum }}r_{k}\gamma^{k}$, where *γ* ∈ [0, 1] is a discount factor and *r*_*k*_ the reward value at step *k*.

In our work, to optimize the criterion, we train a deterministic policy π : *S* → *A* jointly with the state-action value function Q in an actor-critic set-up:

(1) $$\begin{array}{r} Q_{\pi }\left(\text{s},\text{a}\right)=E_{\pi }\left[\overset{\infty }{\underset{k=0}{\sum }}r_{k}\gamma^{k}|\text{s},\text{a}\right],\;\left(\text{s},\text{a}\right)\in S\times A. \end{array}$$

#### 2.1.2. Deep reinforcement learning

The curse of dimensionality (Bellman, 1961), the problem of representing RL functions with a large input space was explored with neural networks a long time ago (Tesauro, 1994). However, the use of neural networks for RL became more and more popular with the arrival of GPUs and the emergence of deep learning since high-dimensional state spaces could be used without requiring hand-crafted features. For instance, deep autoencoders were used to reduce the state space (composed of raw image pixels) of a Q function (Lange and Riedmiller, 2010) in an unsupervised way. Furthermore, deep convolutional neural networks were utilized to approximate the Q function (DQN: deep Q network) of an agent playing Atari games and outperforming human players (Mnih et al., 2015) directly from raw image pixels.

In our work, we use the DDPG algorithm (Lillicrap et al., 2016) which can solve RL problems with a high-dimensional state space and a continuous action space (like several other candidate algorithms). This algorithm combines the off-policy deterministic policy gradient algorithm (Silver et al., 2014) and the DQN.

DDPG is an “actor-critic” algorithm updating the critic *Q*_*ϕ*_ with parameters *ϕ* and the deterministic policy π_***θ***_ with parameters ***θ*** as follows. At each time-step, we choose a mini-batch of *N*_b_ transitions from a large memory buffer of size *N*_trans_ using a uniform distribution:

$$<s_{i},a_{i},r_{i},s_{i}^{\prime }{>}_{i\in \{1,\ldots ,\;N_{\text{b}}\}}\in S\times A\times \mathbb{R}\times S.$$

The targets of the *Q*_***ϕ***_ neural network are computed using a TD(0) update with a learning rate equal to 1:

(2) $$\forall i\in \{1,\ldots ,N_{\text{b}}\},y_{i}=r_{i}+\gamma Q_{\phi^{\prime }}\left(s_{i}^{\prime },\pi_{\theta^{\prime }}(s_{i}^{\prime })\right).$$

*ϕ*′ and ***θ′*** are the parameters of the target networks updated using a rate parameter *τ* (*t* denotes a time-step):

(3) $$\phi_{t+1}^{\prime }=\tau \phi_{t}+(1-\tau )\phi_{t}^{\prime },\;\theta^{\prime }{}_{t+1}=\tau \theta_{t}+(1-\tau )\theta_{t}^{\prime },$$

The *Q*_*ϕ*_ network updates its weights by minimizing the squared error $\frac{1}{2N_{\text{b}}}\sum_{i\;=\;1}^{N_{\text{b}}}{\left(y_{i}-Q_{\phi }(s_{i},a_{i})\right)}^{2}$. Using target networks greatly contributes to the learning stability of the neural networks and using a memory buffer helps to satisfy the constraint of i.i.d samples for learning with neural networks.

Using the *Q*_***ϕ***_ network and the fact that the policy is deterministic, the following policy gradient is derived:

(4) $$\frac{\partial Q_{\phi }}{\partial \theta }≃\frac{1}{N_{b}}\sum_{i\;=\;1}^{N_{b}}\frac{\partial Q_{\phi }\left(s_{i},\pi_{\theta }(s_{i})\right)}{\partial a}\frac{\partial \pi_{\theta }(s_{i})}{\partial \theta }.$$

This update makes the policy select the actions that maximize the Q function at the batch states. In addition to this algorithm, we use the inverting gradient procedure of Hausknecht and Stone (2016) to bound the actions. This method downscales the gradient when the action computed by the policy approaches its limit. When it exceeds its limit, the gradient is inverted. This mechanism prevents the actions from becoming too large.

### 2.2. Overview

**Figure 1:**
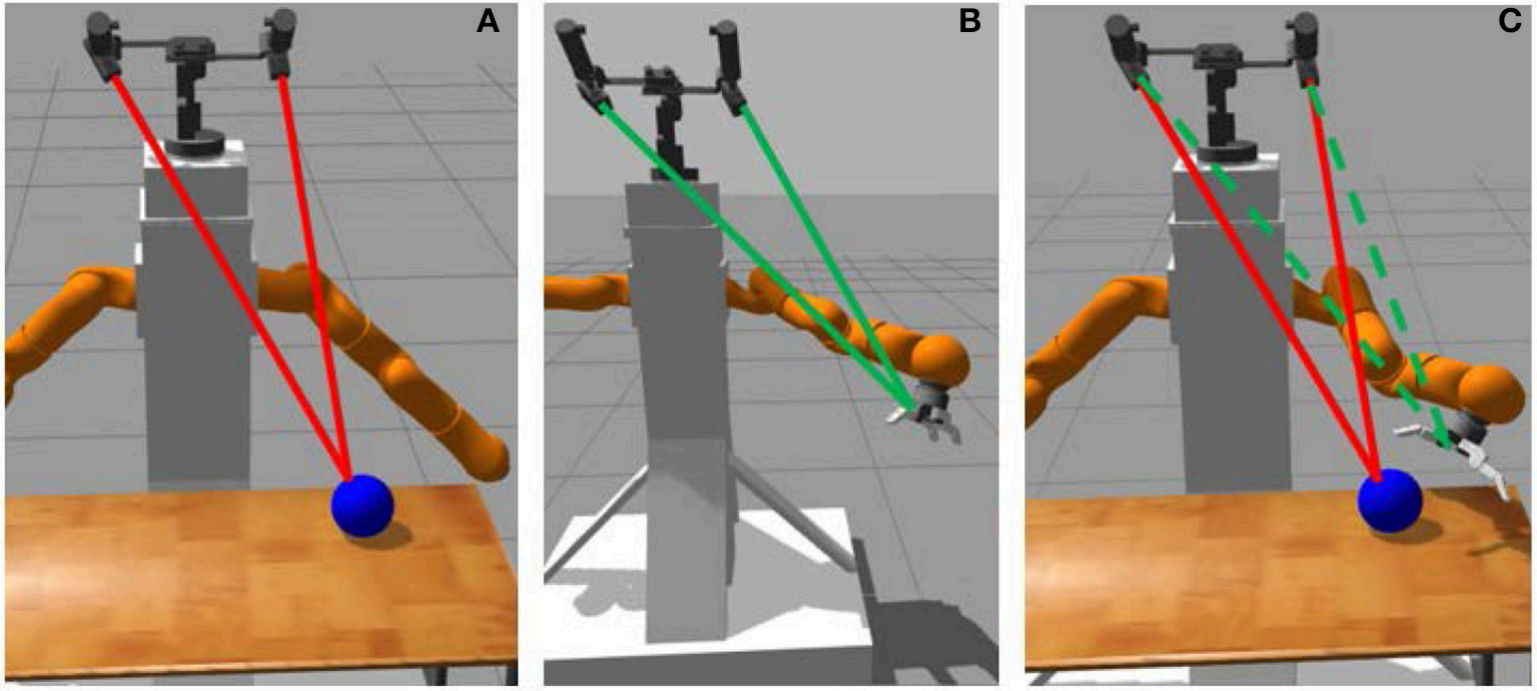
Palm-touching learning process: **(A)** object fixation, **(B)** end-effector fixation and hand-eye coordination, **(C)** palm-touching.

**Figure 2:**
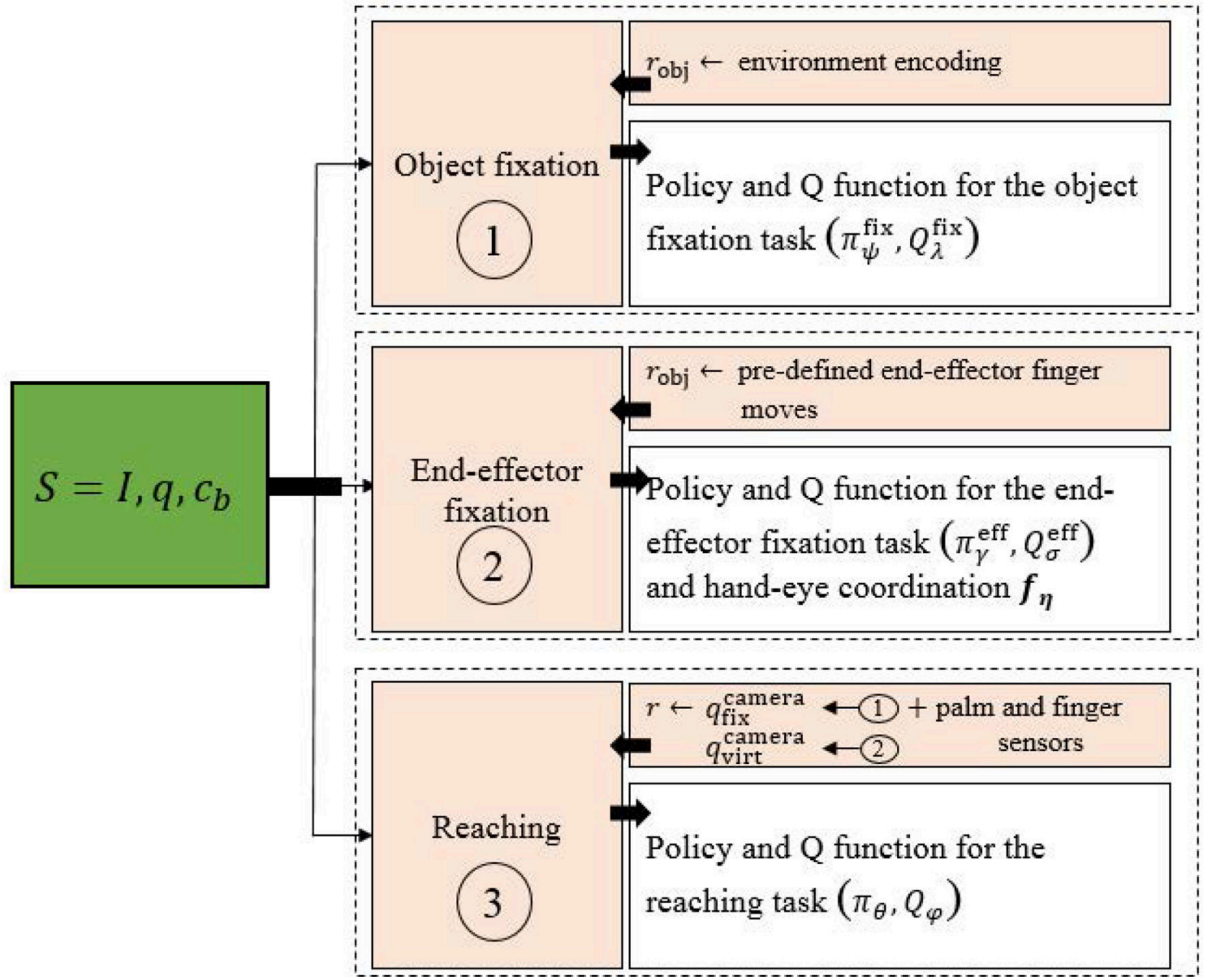
Overall scheme of the touching task learning procedure. Greek subscripts represent neural network parameters.

We describe here the stage-wise learning process (see Figures 1, 2 for a schematic view). For our work, we use a 7 DOF arm with a pair of cameras as shown in Figure 1. The task consists of touching an object on a table with the end-effector palm of the robot. In the following, we use the notations:

- ***I*** = (***I***^left^, ***I***^right^) represents the images from the left and right cameras.
- ***q*** = (***q***^camera^, ***q***^robot^) represents the 3 camera joint angles (one common tilt angle and two independent pan angles) and 7 robot arm joint angles.
- ***c***_b_ is a vector composed of 8 binary values associated with 8 areas of robot fingers. The 8 areas correspond to the proximal, medial and distal areas of the three fingers, with the exception of the proximal area of one finger which is linked to the palm. One binary value becomes 1 when its associated area is in contact with the object and 0 otherwise.

Our stage-wise learning framework is inspired by one of the human ways to locate an object: one can stare an object to locate it. The main objective is to apply this principle with minimal supervision. The proposed method involves three successive tasks:

First, the robot learns from raw pixels to fixate the object with a two-camera system. For this, we use (de La Bourdonnaye et al., 2017) to learn to fixate an object with weak supervision. At the end of the fixation, the camera system coordinates $q_{\text{fix}}^{\text{camera}}$ implicitly encode the object position in 3D space.

Second, the robot learns a hand-eye coordination function *f*_**η**_ which maps robot joint coordinates to virtual camera coordinates:

(5) $$q_{\text{virt}}^{\text{camera}}=f_{\eta }(q^{\text{robot}}).$$

These virtual camera coordinates correspond to the camera coordinates which would make the camera system look at the end-effector. Finally, a reward signal using $q_{\text{fix}}^{\text{camera}}$ and $q_{\text{virt}}^{\text{camera}}$ to make the end-effector close to the object is computed. It is combined with a sparse reward, indicating if the end-effector palm touches the object or not and a term penalizing end-effector contacts with the table (which assumes that the robot has the touching ability to distinguish the object from the table). In the following, we describe each of the three steps.

### 2.3. Learning binocular object fixations

In this part we describe the object fixation learning which the first stage of our method.

#### 2.3.1. Task overview

We define object fixation as bringing the object at the center of ***I***_left_ and ***I***_right_ by moving the cameras. To learn it, we build on our prior work (de La Bourdonnaye et al., 2017), which we summarize below for sake of clarity.

The task is learned with the DDPG algorithm (Lillicrap et al., 2016) using ***I*** and ***q***_camera_ as states, and **Δ*q***_camera_ as actions. The reward function is the sum of left and right camera components: $r_{\text{obj}}=r_{\text{obj}}^{\text{left}}+r_{\text{obj}}^{\text{right}}$. For each camera *cam* = left or right, the reward function $r_{\text{obj}}^{cam}$ is an affine decreasing function of the distance between the image center *x*_*c*_ and the estimated object position $x_{\text{obj}}^{cam}$:

(6) $$\begin{array}{r} r_{\text{obj}}^{cam}=2\frac{\frac{1}{2}d_{\text{max}}-||{\text{x}}_{c}-x_{\text{obj}}^{cam}|{|}_{2}}{d_{\text{max}}}\in \left[-1,1\right], \end{array}$$

with *d*_max_ being the maximal distance between the image center and the object pixellic position.

An episodic set-up is used. For each episode, a random object is put at a random location above the table. The episode ends when a given number of transitions (*N*_e_ = 35) has been reached. Section 2.3.2 describes how *x*_obj_ is obtained with minimal supervision.

#### 2.3.2. Object detection

The object detection mechanism involves a convolutional autoencoder training step in which images of the environment without object are encoded. To do this, we use two 10,000-sized databases (one for each camera) of pictures captured without object in the environment. The camera positions cover a regular grid between joint limits which are set on purpose to keep the table inside the field of view. After the image acquisition, two autoencoders $A_{\mu^{\text{left}}}$ and $A_{\mu^{\text{right}}}$ are trained on each database using ADAM (Kingma and Ba, 2015). Before training, the images are converted for computational purposes from RGB 200 × 200 to 50 × 50 grayscale images.

**Figure 3:**
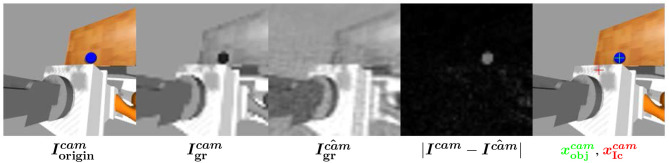
Object detection computation scheme (de La Bourdonnaye et al., 2017).

When the robot is learning to fixate objects, we assume that the object is in the environment. It is detected as an anomaly and localized in the images ***I***^left^ and ***I***^right^. Indeed, we assume objects are badly reconstructed because they are not present in the database images for autoencoder training so that the reconstruction error intensity is higher at the object position. The steps of object detection are presented in Figure 3.

After grayscale conversion and downsampling steps, the autoencoder reconstruction error images |***I***^left^−***Î^left^***| and |***I***^right^−***Î^right^***| are computed (${\hat{I}}^{\text{cam}}=A_{\mu^{cam}}(I^{cam})$). From these error maps, we extract the N points {**x**(*i*)}_*i*∈{1, …, *N*}_ which have the highest intensity. {*L*(*i*)}_*i*∈{1, …, *N*}_ is the set of corresponding luminances. Then, we compute a discrete probability distribution using a kernel density estimator with a Gaussian kernel of zero mean and unit variance:

(7) $$\forall i\in \{1,\ldots ,N\},p(i)=\frac{1}{N}\sum_{j\;=\;1}^{N}L(j)K(x_{i}-x_{j}),$$

with $K(x_{i}-x_{j})=\frac{1}{2\pi }\exp^{-0.5∥x_{i}-x_{j}{∥}_{2}^{2}}$.

After that, the estimated object pixellic positions, respectively $x_{\text{obj}}^{\text{left}}$ and $x_{\text{obj}}^{\text{right}}$ are at the maximal probabilities:

(8) $$\begin{array}{r} x_{\text{obj}}^{cam}=x_{\underset{i}{\arg\max}\left(p\left(i\right)\right)} \end{array}$$

This object detection principle only requires an autoencoder pre-training step without object and the assumption that there is an object in the scene subsequently. Note that the potential noise of this object detection has been tackled using a learning method in de La Bourdonnaye et al. (2017).

### 2.4. Learning a hand-eye coordination function *f*_**η**_

We now describe how a similar framework can be used to learn end-effector fixation and a robot hand-eye coordination function.

#### 2.4.1. Task overview

We model the hand-eye coordination function *f*_**η**_ (see Equation 5) with a neural network. To learn it, we need to have a database *D* of input-output pairs (***q***^robot^, ***q***^camera^) where ***q***^camera^ makes the camera look at the end-effector. To produce such samples, we learn to fixate the end-effector. For this, we use a similar framework as the object fixation task. We use the DDPG algorithm and a reward requiring weak supervision. The Markov Decision Process is the same as for the object fixation with the exception of the reward function. The latter involves the end-effector detection $x_{\text{eff}}^{cam}$ instead of $x_{\text{obj}}^{cam}$:

(9) $$\begin{array}{r} r_{\text{eff}}^{cam}=2\frac{\frac{1}{2}d_{\text{max}}-||{\text{x}}_{\text{c}}-x_{\text{eff}}^{cam}|{|}_{2}}{d_{\text{max}}}\in \left[-1,1\right]. \end{array}$$

The set-up is also episodic. For each episode, random arm joint coordinates are generated using uniform distributions with fixed limits. They are empirically set to provide a large variety of reachable arm configurations.

During learning, training pairs (***q***^robot^, ***q***^camera^) are added to *D* when the reward $r_{\text{eff}}^{cam}$ is above a fixed threshold. When the number of samples in *D* is higher than the batch size *N*_bf_, we train *f*_**η**_ on random batches of *D* each time a new sample is added to *D*.

#### 2.4.2. End-effector detection

We describe here how we detect the end-effector in the image. Unlike de La Bourdonnaye et al. (2017) which uses an autoencoder to localize the object, the end-effector image position is computed using the difference in the image before and after pre-defined end-effector finger moves (Metta and Fitzpatrick, 2003). The idea is that the hand is segmented from the rest of the scene because its appearance varies according to finger moves. Then, this end-effector detection method only requires to specify finger moves.

**Figure 4:**
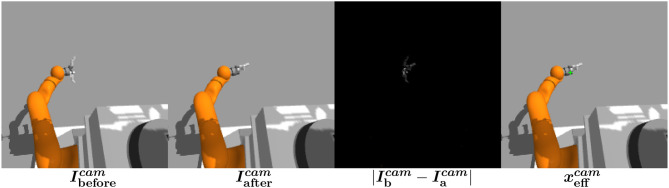
End-effector detection computation scheme.

Figure 4 presents the different steps of the end-effector detection:

- The images before ($I_{\text{before}}^{cam}$) and after ($I_{\text{after}}^{cam}$) the end-effector moves are saved.
- The difference of images is calculated and the end-effector position $x_{\text{eff}}^{cam}$ is computed using a kernel density estimator the same way $x_{\text{obj}}^{cam}$ is calculated from the autoencoder reconstruction error image.

Note that the end-effector detection is also filtered as in section 2.3.2.

### 2.5. Learning to touch

In this section, we describe how the previous learned tasks help to learn to touch the object.

#### 2.5.1. Task overview

**Figure 5:**
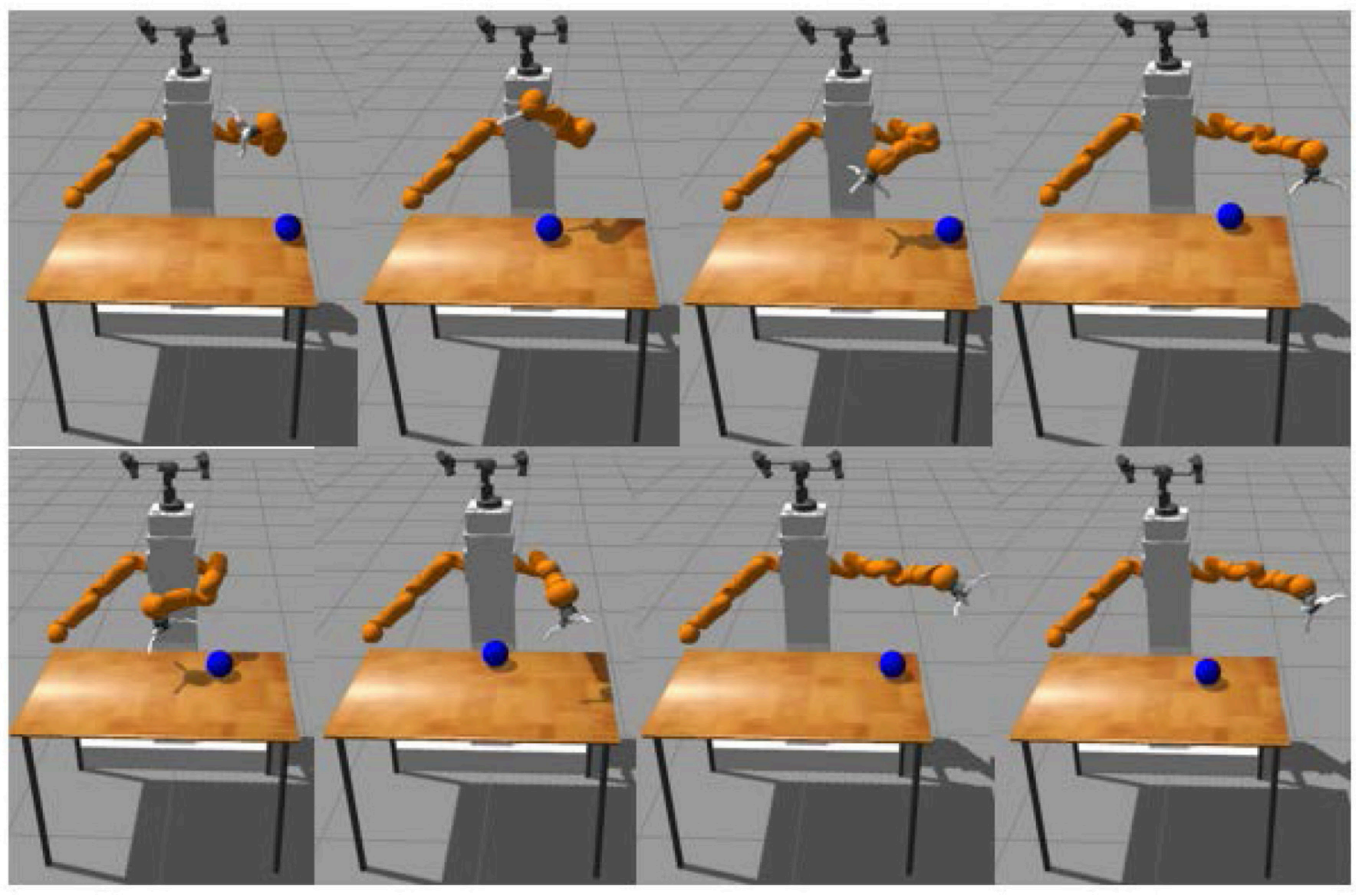
Random examples of initial configurations.

The touching task consists of reaching with the end-effector palm the object above the table at different reachable positions from a large set of initial arm positions (see Figure 5 for a display of 8 randomly generated initial configurations). The goal of learning to reach both at different target positions and from a large set of initial robot joint angles is mainly motivated by the fact it allows to learn policies that are more robust to perturbations in the joint space (Rajeswaran et al., 2017). In addition, reaching from different initial joint angles allows to reach from positions with a badly oriented end-effector which is a challenging task.

The objective of the task is defined by a sparse reward term *r*_sparse_ which indicates if there is palm-touching or not:

(10) $$r_{\text{sparse}}=\{\begin{array}{ll} 1, & \text{if success,}\\ p_{\text{time}}\in {\mathbb{R}}^{-}, & \text{otherwise}. \end{array}$$

Note that the negative term *p*_time_ ensures that the robot looks for the quickest path to the goal. The state space *S* is composed of the arm and camera joint angles ***q*** as well as eight binary tactile sensors **c**_b_ attached to the fingers of the Barrett Hand. Images are not required here because we use a single object and consider that the camera joint angles give sufficient information about the 3D object position. However, they would be necessary if objects with different shapes were used in the experiments. The actions are variations of the robot joint angles: ***a*** = ***Δq***^robot^ which are seven real-valued scalars.

#### 2.5.2. Reward computation

To compute the touching reward function, we use the object binocular fixation policy and the hand-eye coordination function. After the execution of an object fixation step (using the object fixation policy ***π_ψ_***), we get the fixation camera angles ${\text{q}}_{\text{fix}}^{\text{camera}}$ which implicitly encode the object 3D position. After that, using Equation (5) at each time-step, the hand-eye coordination function *f*_**η**_ gives us ${\text{q}}_{\text{virt}}^{\text{camera}}$ which implicitly encodes the end-effector 3D position. Then, a reward shaping term *r*_shCam_ can be computed:

(11) $$r_{\text{shCam}}=\{\begin{array}{ll} 0, & \text{if success,}\\ c_{\text{cam}}∥q_{\text{fix}}^{\text{camera}}-q_{\text{virt}}^{\text{camera}}{∥}_{2}-p_{\text{time}}, & \text{otherwise}. \end{array}$$

with $c_{\text{cam}}\in {\mathbb{R}}^{-}$. *r*_shCam_ represents an informative term which depends on the distance between the virtual camera coordinates and the camera coordinates which make the camera system fixate the object. Thus, it encourages the end-effector to be close to the object. Note that the slope *c*_cam_ is chosen to ensure shaping rewards are small compared with the non-zero sparse reward.

Using these sole terms yields decent performances but we observed that the robot was badly guided when it is close to the table. Indeed, fewer moves are physically plausible and the algorithm can take time to learn them. To accelerate this selection, we propose a new tactile reward term *r*_penContact_ to the reward function penalizing states where the end-effector is in contact with the table:

(12) $$r_{\text{penContact}}=\{\begin{array}{ll} p_{\text{contact}}\in {\mathbb{R}}^{-}, & \text{if contact between the}\\ & \text{end-effector and the table,}\\ 0, & \text{otherwise}. \end{array}$$

Indeed, by applying penalties, we hope that the robot explores areas where it is not in contact with the table, i.e., where it can move without too many constraints to the goal. To compute this term, we make the assumption that the robot knows from its tactile sensors whether it is touching the table.

Finally, the reward function is built from the three previous terms:

(13) $$\begin{array}{r} r_{\text{proposedPen}}=r_{\text{sparse}}+r_{\text{penContact}}+r_{\text{shCam}} \end{array}$$

The relative effect of each of these terms is evaluated in the experiments.

### 2.6. Experiments

In this section, we describe the experimental protocol. The objective of experiments is to evaluate learning performances using the proposed reward function and other ones because they allow to evaluate the relative impacts of each reward term. Besides, we wish to evaluate whether our weakly supervised reward can reach same performances as with a supervised counterpart.

#### 2.6.1. Different reward functions

The reward functions which will be used in our experiments are listed below:

- $r_{\text{sparse}}=\{\begin{array}{ll} 1, & \text{if success,}\\ p_{\text{time}}, & \text{otherwise}. \end{array}$
  This reward is described by Equation (10) and rewards the robot only when the palm touches the object. Besides, it penalizes each unsuccessful movement to encourage the robot to quickly touch the object. Note that using such a sparse reward means that we only dispense with the hand-eye coordination information. Indeed, information brought by the object fixation (the camera joint angles) is still present in the state space.
- *r*_proposedPen_ = *r*_sparse_+*r*_penContact_+*r*_shCam_
  This is the proposed reward function (described in Equation 13).
- *r*_proposed_ = *r*_sparse_+*r*_shCam_
  This is the proposed reward function without the penalties for the contact between the end-effector and the table. This is used to show the influence of the penalty in the learning procedure.
- *r*_sparsePen_ = *r*_sparse_+*r*_penContact_
  We add to *r*_sparse_ a term penalizing contacts of the end-effector with the table.
- *r*_supervisedPen_ = *r*_sparse_+*r*_penContact_+
  $\{\begin{array}{ll} 0, & \text{if success,}\\ c_{\text{cart}}p-p_{\text{target}}{∥}_{2}+0.0125, & \text{otherwise}, \end{array}$
  with *c*_cart_ < 0. To build this reward, we give a 3-dimensional end-effector target Cartesian pose **p**_target_ for the shaping part and we add a sparse reward as well as a term penalizing end-effector contacts with the table. This reward is the closest to the proposed *r*_proposedPen_ but its shaping term requires forward kinematics and 3D object pose information. Finally, the slope *c*_cart_ is chosen to make the shaping term take about the same values as *r*_shCam_.

Note that we choose not to compare our reward function with a Cartesian shaping reward without a sparse term. Indeed, for such a reward function, a success would be to touch with the palm from a specific orientation and position. In our case, a success can be to touch with the palm in any position. The tasks are then too different to be compared in terms of touching improvement.

#### 2.6.2. Material

We describe here the material we use for our experiments.

**Figure 6:**
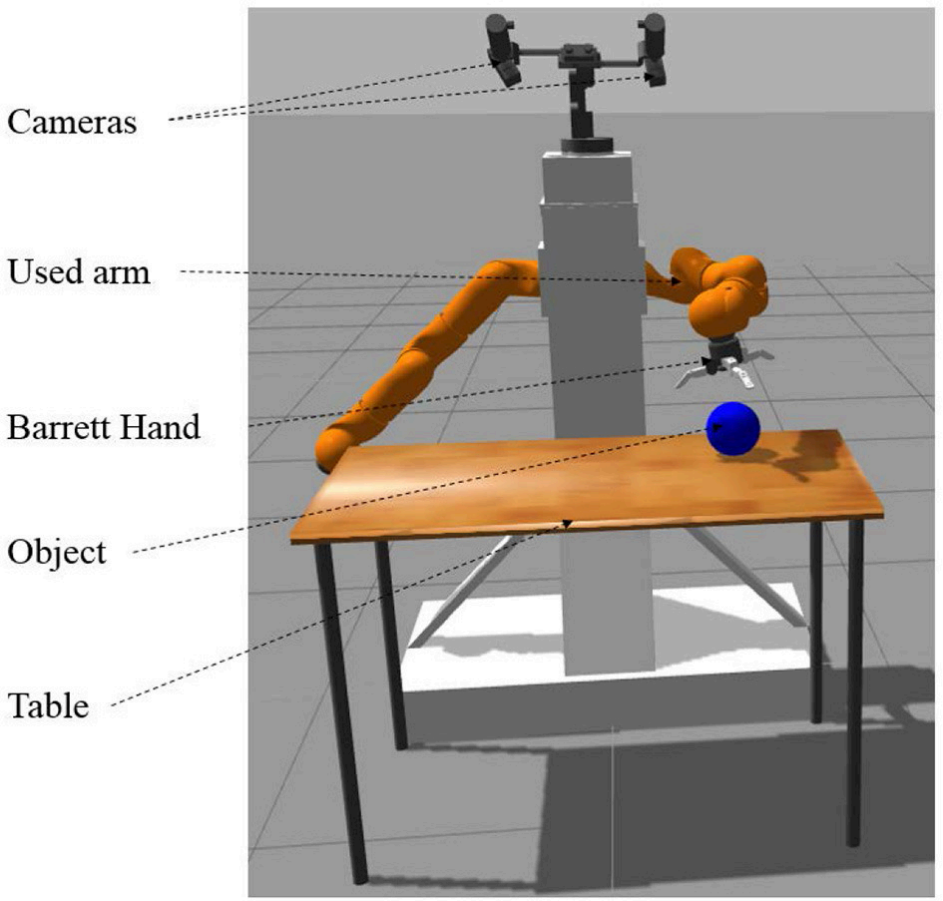
Scheme of the robotic platform.

Figure 6 presents the chosen virtual experimental platform which is the realistic representation of one of our real robotic platforms. The simulations use the Gazebo simulator with the ROS (Quigley et al., 2009) middleware. The robotic platform is composed of three entities:

- A two-camera pan-tilt system attached above the platform
- Two robotic arms attached on the left and right sides of the platform. Note that we use only one arm in our experiments.
- A Barrett hand is attached to the arm that we use in the experiments.

**Figure 7:**
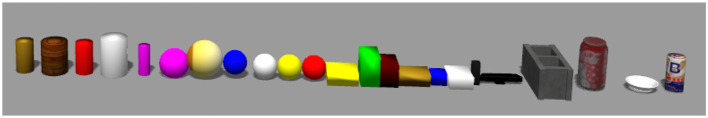
Training set for the object fixation task (de La Bourdonnaye et al., 2017).

A table from the Gazebo object database is placed below the cameras and in front of the bi-arm platform. This table is not present when we learn the hand-eye coordination function. To learn object fixation (de La Bourdonnaye et al., 2017), we use some objects from the gazebo object database and several hand-made ones with various shapes and colors (see Figure 7). We use a blue-ball for the reaching experiments though the method does not depend on this specific model since the robot learns to look at any object.

#### 2.6.3. Experimental protocol

We describe how we compare the policies learned with different reward signals for the touching task. The protocol contains a training and a test phase.

##### 2.6.3.1. Training phase

For training, we use the DDPG algorithm (Lillicrap et al., 2016) and the previously defined reward functions. Learning happens on *N*_tot_ bounded-length episodes of maximal size *N*_max_. Each episode has an initial arm position and an object position. The object position is uniformly chosen from a rectangular area of reachable object positions on the table. The initial robot joint angles are sampled from a set of uniform distributions (each one corresponding to a robot joint angle). When an initial position leads to a collision between the arm and its environment, the initial position is re-set until a collision-free position is sampled.

For the exploration, we use the Ornstein-Uhlenbeck process. This correlates the noise ϵ_*j*_(*t*) for a joint at time *t* with the noise ϵ_*j*_(*t* − 1) of the same joint at time *t*−1 with the equation:

(14) $$\begin{array}{r} \epsilon_{j}\left(t\right)=\theta_{j}\mu_{j}+\left(1-\theta_{j}\right)\epsilon_{j}\left(t-1\right)+\left(\xi_{j}\left(t\right)\sim N\left(0,\sigma_{j}\right)\right). \end{array}$$

*θ*_*j*_ is a factor trading-off the correlation with the previous noise and the correlation with the equilibrium value *μ*_*j*_ and *σ*_*j*_ is the standard deviation of the used Gaussian distribution. This exploration procedure is particularly interesting in problems in which the same action applied during several time-steps can be the optimal behavior.

As the task requires a precise orientation of the end-effector, the robot frequently blocks itself close to a reaching position. For instance, the robot can touch the object with its fingers without touching it with the palm. And, if the actions computed by the policy make the end-effector move downward, the robot can be blocked by the table despite exploration. Thus, to avoid these situations, we handle the times when the robot is blocked without succeeding in reaching. More precisely, when the robot is blocked a backward action is taken, i.e., the robot goes back to a previous contact-less position. This allows to more correctly discriminate actions in the contact areas in the sense the robot is provided with other chances of success.

Through the training experiments, we wish to compare our reward requiring little supervision with other ones. Consequently, for all the reward signals, in order to monitor the learning progress, we specifically plot the reaching frequency ν^reward^ over the episodes, reward referring to a specific reward function. We average six experiments per setting and provide confidence interval plots [*m*^reward^, *M*^reward^] for each computed average. *m*^reward^ and *M*^reward^ are computed according to the equations below:

(15) $$\begin{array}{r} m^{\text{reward}}=\nu^{\text{reward}}-\frac{1.96\sigma^{\text{reward}}}{\sqrt{N_{\text{run}}}}, \end{array}$$

(16) $$\begin{array}{r} M^{\text{reward}}=\nu^{\text{reward}}+\frac{1.96\sigma^{\text{reward}}}{\sqrt{N_{\text{run}}}}, \end{array}$$

with *N*_run_ being the number of runs per reward function and σ^reward^ the standard deviation of the reaching frequency for each reward function.

Furthermore, we record *N*_1_ the number of episodes it took to reach a first reaching success, *N*_90_ the number of RL iterations it took to reach and remain above reaching performance of 90 % as well as associated confidence intervals and standard deviations. These variables are used to evaluate the learning velocity with different reward functions.

##### 2.6.3.2. Test phase

To evaluate the learned policies, we apply them without any exploration noise on *N*_tot_ random episodes and we compute the touching frequency $\nu_{\text{test}}^{\text{reward}}$. Moreover, for each reward signal, we provide a confidence interval for the average touching frequency and the standard deviation of the touching frequency $\sigma^{\nu_{\text{test}}^{\text{reward}}}$. Note that we do not apply the systematic backward motion used in the training phase to deal with blocked situations. Instead, when the robot is blocked, it just follows the learned policy. Like in the training phase, the results are averaged over six experiments per setting.

#### 2.6.4. Implementation details

For all the neural network algorithms, we use the caffe library (Jia et al., 2014). A GPU (nvidia GeForce GTX Titan X) is used for the experiments.

We use the same neural network architectures as in de La Bourdonnaye et al. (2017) for the end-effector and object fixation tasks. The hyperparameter values are also the same with the exception of the number of iterations: 200, 000. The hand-eye coordination function is a neural network with 2 fully connected hidden layers of 10 and 5 neurons and a batch size *N*_bf_ of 32 is used to learn it. For the episode initialization of the end-effector fixation task, the seven arm joint angle distribution amplitudes are 11, 46, 69, 92, 92, 86, and 0° if we consider the ascending order in the kinematic chain i.e., from the base link to the end-effector.

For the touching task, the Q network has 3 fully connected layers with 250, 200, and 1 neural units. The policy network involves 3 fully connected layers with 200, 150, and 7 neural units. The weights are updated using the Adam solver (Kingma and Ba, 2015). Tables 1, 2 provide values for the parameters used in the experiments. For the Q update, the discount factor *γ* is equal to 0.99. For the episode initialization, the distribution limits are 23, 57, 80, 91, 103, 80, and 11°.

**Table 1:** Parameter values.

**Parameters**	***N*_max_**	***N*_b_**	***c*_cam_**	***c*_cart_**	***N*_trans_**	***N*_tot_ (training)**	***N*_tot_ (test)**	***p*_contact_**	***p*_time_**
Values	100	256	$-\frac{1}{30}$	$-\frac{1}{40}$	60,000	40,000	1,000	−0.01	−0.0125

**Table 2:** Ornstein-Uhlenbeck process parameters.

**Parameters**	***θ*_*j*_, *j*∈{1, …, 7}**	**μ_*j*_, *j*∈{1, …, 7}**	**σ_*j*_, *j*∈{1, …, 4}**	**σ_*j*_, *j*∈{5, …, 7}**
Values	0.8	0	0.01	0.04

## 3. Results

**Figure 8:**
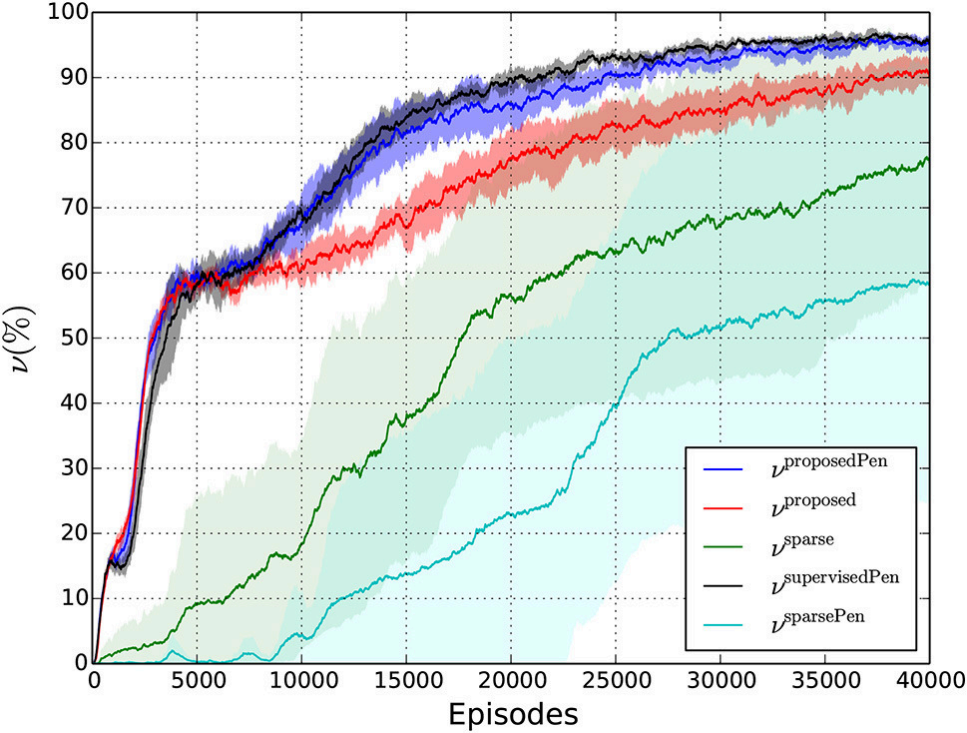
Evolution of the average reaching frequency during training for the different reward functions.

Table 3 presents the final performances of the different policies as well as the number of episodes it takes to get a first reaching success and the number of RL iterations it takes to reach (and remain above) an average reaching performance of 90 %. The average reaching performance is obtained using exponential smoothing: $\nu_{\text{f}}^{\text{reward}}=\left(1-\omega \right)\nu_{\text{f}}^{\text{reward}}+\omega \nu_{\text{r}}^{\text{reward}}$, with $\nu_{\text{r}}^{\text{reward}}$ and $\nu_{\text{f}}^{\text{reward}}$ being the raw and smoothed frequencies and ω the smoothing factor being equal to 0.003. Figure 8 shows the experimental training curves as well as associated confidence intervals. Note that this figure use exponential smoothing for visualization purposes. We can notice several important facts:

- Learning the reaching task can work very well because the robot reaches 90% of touching performances with the reward functions using shaping terms (Videos of the policy learned with our reward function can be consulted in the Supplementary Material). This shows that the camera joint angles integrated in the state space encode sufficiently well the object position, which confirms the rationale of our method.
- With the use of sparse-only rewards, the probability of getting the first success is low. It takes a lot of episodes to reach a first success (from 7,505 episodes for the *N*_1_ values). Furthermore, we cannot have a precise idea about the time when the first success occurs because the standard deviations are very high. Moreover, *N*_90_ values are not available for these two reward settings because some of the runs were not successful at all. Finally, as shown by Figure 8, the confidence intervals for the average reaching frequency are very large, which means that the average estimation is not precise at all for the sparse reward settings. The only fact we can notice for these settings is that it can work for a run and totally fails for another one. Then, these reward functions do not ensure a reliable learning.
- With a shaping term, the probability of having first successes is much higher. The different *N*_1_ values for *r*_proposedPen_, *r*_proposed_, *r*_supervisedPen_ are of the same order of magnitude, are small, and exhibit low standard deviations. And our weakly-supervised setting allows to approach similar reaching performances compared with its supervised counterpart even if the final reaching frequency is slightly lower. In addition, Figure 8 shows that the confidence intervals of ν^supervisedPen^ and ν^proposedPen^ intertwine even if the bounds of ν^supervisedPen^ are generally higher. This shows that even if ν^supervisedPen^ is higher than ν^proposedPen^ most of the time, results are close. Furthermore, we notice that three phases can be distinguished. From 0 to about 5,000 episodes, the reward curves increase with the same velocity. It corresponds to a phase in which some initial positions are mastered without substantial end-effector orientation changes. Indeed, for some initial positions, the robot has to change only a little its end-effector orientation to reach a grasping posture. After 5,000 episodes, there is a period of slow increase for the three settings and from about 7,000 episodes, “harder” initial positions are more and more mastered. We observe that the three settings start to distinguish from each other and the term penalizing contacts seems to be a decisive factor.
- Indeed, with a term penalizing contacts between the end-effector and the table, learning becomes faster. To show this, we can compare *r*_proposedPen_ and *r*_proposed_: *N*_90_ is lower for *r*_proposedPen_, $\nu_{\text{test}}^{\text{proposedPen}}$ is higher than $\nu_{\text{test}}^{\text{proposed}}$, and Figure 8 shows that ν^proposedPen^ is always superior to ν^proposed^ after 10,000 episodes. Furthermore, in Figure 8 we notice that the upper bound *M*^proposed^ is generally inferior to the lower bound *m*^proposedPen^. All of these observations show the supremacy of *r*^proposedPen^ over *r*^proposed^. It shows that penalizing contacts between the end-effector and the table has an important influence on learning performances. The reason is that it is easier to experiment “good” moves in contact-less areas given the robot can easily be blocked when it touches the table.

**Table 3:** Values featuring learning velocity (*N*_1_ and *N*_90_), final reaching frequency ( $\nu_{\text{test}}^{\text{reward}}$) and associated standard deviations.

**Reward**	***r*_proposedPen_**	***r*_proposed_**	***r*_supervisedPen_**	***r*_sparsePen_**	***r*_sparse_**
*N*_1_	95 ± 16	110 ± 19	105 ± 29	7505 ± 6918	8214 ± 4145
*N*_90_	(1.73 ± 0.25) × 10^6^	(2.35 ± 0.17) × 10^6^	(1.55 ± 0.13) × 10^6^	N/A	N/A
$\nu_{\text{test}}^{\text{reward}}$ (%)	94.9 ± 1.3	90.7± 2.83	97.2 ± 0.67	59.9 ± 37.6	81.9 ± 17.3
$\sigma^{N_{1}}$	20	23	36	8646	5180
$\sigma^{N_{90}}$	312,911	172,129	165,160	N/A	N/A
$\sigma^{\nu_{\text{test}}^{\text{reward}}}$ (%)	1.64	3.53	0.84	47	21.6

## 4. Discussion

### 4.1. Contributions

Our first contribution is the design of a stage-wise learning framework to learn a complex reaching task. This framework involves the DDPG algorithm though any deep RL algorithm suitable to continuous action spaces could be used. Interestingly the first two tasks are largely similar in their modeling: we use the same MDPs with the exception of the reward function, the same kernel density estimator for localizing the point of interest in the image and the same filtering method to remove the detection noise. The knowledge of the two tasks are then combined to compute an informative shaping reward efficiently guiding the robot toward reaching postures.

Our second contribution is to learn the task with only weak supervision, i.e., without kinematics, calibration or pre-processing blocks and to exhibit similar performances compared with a fully supervised reward function. Furthermore, our framework is applied on a challenging task with a large set of initial configurations: several initial arm positions and several object positions as shown in Figure 5.

### 4.2. Related work

Our approach resembles some developmental robotics methods which learn to reach using a hand-eye coordination function and object fixation. However, they are usually paired with supervision for the object and/or end-effector fixations (Nori et al., 2007; Chinellato et al., 2011; Jamone et al., 2012; Law et al., 2014) or computation of action primitives (Hoffmann et al., 2005). In other terms, object or end-effector detection use markers or simple blob-detection algorithms which would not be valid for any kind of object. The contributions brought by these papers are more related to the learning architecture which is close to the one of infants whereas our work focuses on reducing the amount of external information used for learning.

The multi-target-position multi-initial-arm-position setting has also been implemented on a simulated reaching task using a 7 DOF manipulator (Lillicrap et al., 2016). However, there were neither collision aspects nor orientation constraints for the end-effector and a supervised shaping reward was used. Lampe and Riedmiller (2013) learns an object grasping policy but integrates the object position in a camera image in a relatively low-dimensional state space, which requires a supervised visual module. Popov et al. (2017) learns a brick grasping task from several initial arm positions at several target positions. However, for the arm initialization, the end-effector is always made close to the object and its orientation adapted to a grasping action.

### 4.3. Limitations

Our approach has certain limitations mainly related to the first stages of the stage-wise framework. In the object fixation step, even though learning is weakly-supervised, if the environment varies, the approach in its current form needs the intervention of a human user to learn again to encode the environment. Besides, our approach constrains objects not to be present in the scene when the environment is encoded. And finally, the fixation cannot be applied on the object when the arm is above the table. Concerning the hand-eye coordination learning stage, the method implemented here requires an immobile background to make the end-effector detection method work. Note that this problem is solved in the literature by correlating finger moves with detection changes in the image Metta and Fitzpatrick (2003).

## 5. Further research

As further research, we wish to make the first and the second stages of our framework robust respectively to environment variations and moves in the background and also to be able to fixate the object when the arm is above the table. A good hint for this would be to achieve an open-ended learning framework in which the learnings of the tasks presented in the paper overlap and drive each other. For example, learning to reach an object with the end-effector may first help the robot to acquire the knowledge of what is an object and would consequently drive the learning of object fixation. Second, it may help the robot to acquire hand-eye coordination.

Furthermore, it would be interesting to learn other kinds of manipulation tasks including complex ones such as inserting a key in a lock with our framework. In principle, switching from a task to another one would just require to switch from a sparse reward to another one. However, some sparse rewards are less likely to be reached that other ones, e.g., grasping is less likely than palm-reaching. Consequently, our learning framework might not be directly applicable for too complex tasks, and learning them with our framework would be achieved by learning a curriculum of tasks, from the simplest to the most complicated. Finally, we wish to adapt the framework to a real robotic setting.

## Author contributions

FdLB implemented the code and wrote the original version of the paper. JT, TC, and CT gave advice on the work itself and reviewed the paper.

### Conflict of interest statement

The authors declare that the research was conducted in the absence of any commercial or financial relationships that could be construed as a potential conflict of interest.

## Supplementary material

Video S1 Video showing the different learned behaviors from object fixation to reaching.
